# The Process of Nanostructuring of Metal (Iron) Matrix in Composite Materials for Directional Control of the Mechanical Properties

**DOI:** 10.1155/2014/979510

**Published:** 2014-02-17

**Authors:** Elena Zemtsova, Denis Yurchuk, Vladimir Smirnov

**Affiliations:** Saint Petersburg State University, Universitetskii Pr. 26, Saint Petersburg 198504, Russia

## Abstract

We justified theoretical and experimental bases of synthesis of new class of highly nanostructured composite nanomaterials based on metal matrix with titanium carbide nanowires as dispersed phase. A new combined method for obtaining of metal iron-based composite materials comprising the powder metallurgy processes and the surface design of the dispersed phase is considered. The following stages of material synthesis are investigated: (1) preparation of porous metal matrix; (2) surface structuring of the porous metal matrix by TiC nanowires; (3) pressing and sintering to give solid metal composite nanostructured materials based on iron with TiC nanostructures with size 1–50 nm. This material can be represented as the material type “frame in the frame” that represents iron metal frame reinforcing the frame of different chemical compositions based on TiC. Study of material functional properties showed that the mechanical properties of composite materials based on iron with TiC dispersed phase despite the presence of residual porosity are comparable to the properties of the best grades of steel containing expensive dopants and obtained by molding. This will solve the problem of developing a new generation of nanostructured metal (iron-based) materials with improved mechanical properties for the different areas of technology.

## 1. Introduction

In the past two decades scientists have paid much attention to obtaining of composite nanomaterials based on nanoobjects with varying dimensions (nanoparticles, nanofilms, nanowhiskers, etc.) [1, 2]. During such research, for example, in the field of obtaining of structural composite nanomaterials, there was expected that the preparation and use of such materials will dramatically improve mechanical properties of these materials [3–7]. However, in most cases, the effect was not so large compared with the expected [6–8]. There are many reasons, but we can distinguish the main ones:

- necessity for more precise control of nanoobject sizes (preferably in the range of 1–10 nm), for example, the most studied 3D nanoobjects (nanoparticles) which are dispersed phase for the structural composite nanomaterials. This is due to the fact that in most of the proposed methods for the synthesis there are obtained particles characterized by a fairly broad curve of particle size distribution [9–13];
- necessity of development of precise methods for synthesis of a new generation of composite nanomaterials. If staying, for example, on one type of the composite nanomaterials with the metal matrix, the existing methods for producing such materials often include introduction of nanoparticles or nanowhiskers in the molten metal. Almost always particles in the solid (usually with different size) are not evenly distributed in metal matrix volume. This leads to a slight increase in the strength characteristics of composite metal nanomaterials [14, 15].

Thus, when considering the preparation of new generation of nanostructured composite nanomaterials, we should talk about the material [15] in which it is difficult to separate the constituent components of composite materials (matrix and reinforcing filler). In this material reinforcing filler should be a continuous framework (e.g., nanowires) pervading the all volume of matrix frame. This structure consists of two interpenetrating each other continuous frames.

The solution of the above problem (synthesis of highly organized nanostructured materials) involves the creation of material with required level of structural organization. In general, we are talking about artificial “super control” in the solid as an alternative to natural regulation processes, mainly represented by the process of crystallization.

The crystal structure of a solid is characterized by two main parameters: structural units (from a single atom to big molecular objects) and ordering degree (from the amorphous state to monocrystal). Auxiliary parameters are needed for describing artificially synthesized highly organized structures and for characterizing these complex systems. As a rule, the task of any solid-phase synthesis is creation of a certain structure. There are several aspects of understanding the term “synthesis of a solid chemical compound” or its physicochemical analog “synthesis of a certain structure”:

1. the physicochemical aspect includes consideration of composition and crystal (sometimes amorphous) structure;
2. the topological aspect includes consideration of the spatial distribution of the composition and structure (actually, the spatial distribution of structural units of an object);
3. the electronic aspect concerns electronic states of a solid substance under consideration.

The notion of topology implies a complex structure of matter. Therefore, we consider in more detail the notion of topology for highly organized solid compounds. This notion is related to the possibility of various spatial atomic distributions in an artificially synthesized substance [14–16].

**Figure 1:**
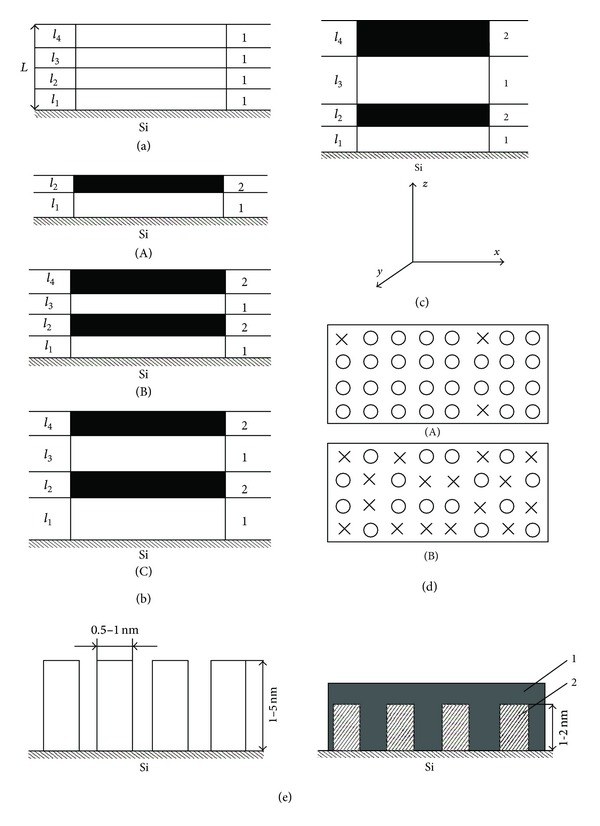
Scheme of two-dimensional nanostructures at silica surface. (a) Uniform distribution of chemical composition and structure, for example, monolayers of element-oxygen groups of a given composition: (*l*) monolayer thickness; (*L*) total layer thickness, *l*_1_ = *l*_2_ = *l*_3_ = *l*_4_; and (1) Ti-O monolayers. (b) Periodical distribution along the *z*-axis of element-oxygen layers containing a certain number of monolayers: (1) Fe-O groups and (2) Ti-O groups ((A) two-layer structure, *l*_1_ = *l*_2_ and (B) four-layer structure, *l*_1_ = *l*_3_ and *l*_2_ = *l*_4_). (c) Aperiodic distribution of element-oxygen layers along the *г*-axis (four-layer structure with *l*_1_ ≠ *l*_2_ ≠ *l*_3_ ≠ *l*_4_). (d) Aperiodic atomic distribution in the surface monolayer plane (top view): (crosses) Fe-O groups and (circles) Ti-O groups ((A, B) different Fe-O/Ti-O ratios). (e) Aperiodic distribution of “zero-dimensional” structures in the substrate plane: (1) Fe and (2) Si.

From the viewpoint of topology, the limiting states are, on one side, a substance uniform in composition and structure (e.g., monocrystal, glass, etc.) and, on the other side, multielement (not in a chemical, but in a structural meaning) structure where each structural unit can be arbitrarily connected with others (e.g., biological objects). Artificial structures of the simplest topological organization (uniformly laminated structures, Figure 1(a)), for example, with thin oxide layers (*L* > 30 nm), have long been prepared [14]. A more complex topological organization includes nonuniform distribution of composition and structure (layered structure) along one of the spatial coordinates both of periodic (a) and of aperiodic (b, c) character. The synthesis of such structures is well run. However, certain difficulties arise if it is necessary to obtain a small nanostructure period (*L*) of order 1 nm.

Further complication of topological organization is possible on organizing atoms also in the monolayer plane (for one or several monolayers) (Figure 1(d)). Still higher levels of organization of matter imply formation of spatial nonuniformity in a structure synthesized and differ in the degree of internal symmetry (Figure 1(e)).

In fact, we discuss the possibility of realizing the processes of superordering in a solid, that is, formation (synthesis) of ordered distributions of matter of a certain size. Let us compare the realization of natural ordering and artificial superordering in a solid. It is of note that macroscopically ordered distributions are sufficiently widespread in nature. As examples, macroscopic ordered distributions in plumeous clouds, in the radial structure of volcanoes, in ordered eutec-tics, and in sea sandbanks can be enumerated.

Since these examples are related to the structure of matter, the processes under consideration involve mass transfer (diffusion) and may follow the only direction to a uniform state of the system. The origin of such processes lies in that they are caused by thermodynamic driving forces and directed to extreme values of thermodynamic parameters, including the entropy maximum at equilibrium.

The closer a system to equilibrium, the more pronounced are internal self-ordering processes in the system, the easier is maintaining physicochemical conditions, and the more difficult is changing them by external action. Hence, as we approach equilibrium, the range of allowed nonuniform structures becomes narrower, while uniform structures become simpler to synthesize. The reverse situation takes place far from equilibrium: a very precise external control is needed, but almost all structures are allowed. The choice of an optimal synthetic strategy is one of the most intricate problems.

Thus, the only way to create an aperiodic order consists in changing the ordering processes described by equilibrium thermodynamics and directed to securing system stability by a synthetic program directed to obtaining highly organized solid-phase structures in the metastable state.

Because of the low mobility of atoms in a solid, a certain thermodynamically unfavorable structure (e.g., diamond) can exist for any long time.

It is now firmly established that the synthesis of solid chemical compounds by crystallization can result in formation, from the same structural units, of a number of equivalent variants of composition and structure with close energies (actually, this is an unseparable mixture of variable composition). This occurs since the solidification process always proceeds at a certain supersaturation [17]. In the case of the solid-phase synthesis of highly organized structures, a solid compound to be obtained should possess the following characteristics [18]: (a) any topology including uniform and nonuniform distribution of composition and structure should be attainable; (b) aperiodic structure (periodicity is possible as a particular case); and (c) thermodynamic nonequilibrium state. We imply that the chemical potentials in the existing homogeneity regions are different under chosen conditions (temperature, pressure, etc.) and this does not lead to destruction of the resulting structure.

It is of note that chemical processes on the atomic-molecular level should play the main role in the synthesis of nanostructures with a small period and a given topology. Then all the processes are reduced to a certain sequence of surface chemical reactions between functional groups of the solid and molecules of necessary chemical nature [19]. Herewith, the only way for creating highly organized structures (of aperiodic order) is replacing crystallization by the synthesis of solid-phase structures in the metastable state. In fact, to accomplish such synthesis, one should control the activity of some chemical bonds and prevent reaction of other chemical bonds.

There is an important aspect to be used at realizing the solid-phase matrix synthesis. With microcrystal seeds, the main role is played by the surface of a solid, since physicochemical processes just develop at the surface. It is of note that thermodynamic and kinetic barriers are not eliminated but only diminished by introduction of a seed (substrate) in a system. Separate relative equilibrium is established for each substrate under given conditions. Traditional methods of epitaxial synthesis (deposition) are controllable processes. However, it is hard to speak about obtaining a nanometric substance layer of a given thickness by the epitaxial deposition method because epitaxial layers are formed by way of appearance and integration of crystal nuclei whose dimensions certainly exceed the monolayer thickness.

Additional difficulties in realizing the synthesis of solid substances arise from the fact that the overwhelming majority of reactions used in chemical science and technology belong to the type of “unorganized” reactions. We mean the reactions with particles (atoms, molecules, ions, or radicals) reacting at accidental meetings both in time and in space and by mutual orientation. In other words, there is no spatiotemporal molecular and solid-phase organization of chemical interaction in such reactions. At the same time, the possibility of such an organization is known from molecular biology, for example, the biosynthesis process [20]. For example, Merrifield has realized the solid-phase (matrix) synthesis of polypeptides [21, 22] by successive extension of the polypeptide chain on an inert polymeric substrate.

The problem of a standard solid surface remains actual in synthesis of nanostructures. Let us consider the principles of synthesis of solid substances of reproducible composition [23, 24]. They are the process irreversibility conditions, the availability of a matrix, and the program of production and accumulation of information.

When performing controlled solid-phase synthesis, a researcher actually carries out a number of operations to obtain a solid chemical substance possessing (a) a certain excess of free energy contained in the system of interatomic bonds, as compared with the initial reagents, and (b) a higher degree of ordering of interatomic bonds as compared with the initial reagent mixture at equilibrium.

Practically, the reproducible synthesis of nanostructured solid substances is realized via chemical assembling of structural units on appropriate matrices. The monolayer-by-monolayer chemical assembling of solid substances involves a number of preprogrammed surface reactions with at least bifunctional molecules of either one substance or another.

The principal significance of this method of synthesis of solid matter is “that, instead of spontaneous packing structural units in the crystallization process, their forced packing in a preset order is performed, that is, preset composition and structure of the solid substance are realized” [23]. By varying temperature and other conditions of chemical assembling, one can change the density of packing of structural units. Apart from crystalline structures, one can create other, significantly more complex structures whose number has no limit.

We now turn to chemical reactions performed in the process of synthesis. The progress in the field of precise inorganic synthesis is associated with the rapid development of surface chemistry. This branch of the inorganic chemistry of solid substances studies chemical reactions in the surface atomic layer of a solid substance and also studies the ways controlling these reactions [15]. Such reactions open the unique possibility for controlling, on the atomic level, the synthesis of new substances of reproducible composition and structure by means of reactions between functional groups of a solid substance and molecules of necessary chemical nature. Such surface reactions result in formation of surface chemical compounds.

In order to solve our problem—finding the influence of one-dimensional (nanowires, 1–50 nm) structural heterogeneities basis on TiC, amorphous or crystalline structure on the structure, and functional (mechanical) properties of bulk metallic material (metallic matrix)—it is necessary to solve the problem of substance structuring at the nanolevel. The main theoretical and experimental positions of the chemical nanostructuring are considered in [15]. Herewith surface chemical reactions of forced structuring are the most important phase of nanostructuring. Thus the basic mechanical properties of the resulting solids are provided by the creation of nanostructured metal matrix. The synthesis of nanolayers of the dispersed phase (TiC) was carried out by the method of directed synthesis developed in Russia—molecular layering (ML) [3–6, 13, 25–27], also known abroad under the name “atomic layer deposition” (ML-ALD) [8, 13, 28–34]. Currently this method is recognized by foreign experts as the main method for production of nanolayers in nanoelectronics and in a number of other related areas [9, 10, 25, 35].

Practically, the reproducible synthesis of nanostructured solid substances is realized via chemical assembling of structural units on appropriate matrices. The monolayer-by-monolayer chemical assembling of solid substances involves a number of preprogrammed surface reactions with at least bifunctional molecules of either one substance or another [36].

The main purpose of this work is to establish experimental fundamentals of the synthesis of highly organized nanostructured composite materials with the TiC dispersed phase which will directly regulate the mechanical properties of composite material.

## 2. Materials and Methods

As noted above for the solution of our problem—finding the influence of one-dimensional (nanowires, 1–50 nm) structural inhomogeneities based on TiC on the structure of and functional (mechanical) properties of metal type (iron matrix) bulk material—we should solve the problem of structuring material at nanoscale. In this case, the most important phase of the nanostructuring of matrix is the surface chemical reaction of force structuring. Thus the basic mechanical properties of obtained material are provided by the creation of nanophase dispersion (TiC) in the volume of the metal matrix. Thus, the original metal matrix must be porous. The synthesis of nanolayers of the dispersed phase (TiC) in the pores of the matrix was based on developed in St. Petersburg State University method of directed synthesis—a method of molecular layering (ML), known abroad under the name “atomic layer deposition” (ML-ALD).

Process of preparation of nanostructured metal material includes a number of successive steps: (1) preparation of nanoparticles of iron oxide; (2) reducing iron oxides (by hydrogen or other reducing agents) in order to obtain ultrafine iron metal frame with a predetermined porosity; (3) surface nanostructuring of prepared metal porous matrix by TiC nanolayers (1–50 nm); (4) consolidation (pressing) and sintering to obtain a bulk (nonporous) material with nanostructured metal material with size of TiC nanowires (1–50 nm). The third step of the synthesis involves a fundamentally new scientific approach for the development of scientific bases of ultraprecise synthesis, that is, combined chemical and physical processes leading to the creation of a given order of the atoms in synthesized structure or nanostructured material.

Following each cycle of surface chemical reactions (SCR) carried out under strictly controlled conditions on the solid surface, there is a deposition of monolayer of new structural building units (carbides) chemically associated with the original substrate and the “thickness” of a monolayer is 2-3 Å. Carrying out a number of these reactions a layer of substance with a certain thickness can be synthesized.

Chemicals and materials were obtained from commercial suppliers: TiCl4 (99.6%), (Alfa Aesar), carbon tetrachloride (99%) (Sigma-Aldrich), FeSO_4_·7H_2_O, NaOH, HNO_3_ (Merck), and CH_4_ (gas) (Lengaz).

### 2.1. Preparation and Reduction of Iron Oxide in Order to Obtain Ultrafine Iron Metal Frame with a Given Porosity

**Figure 2:**
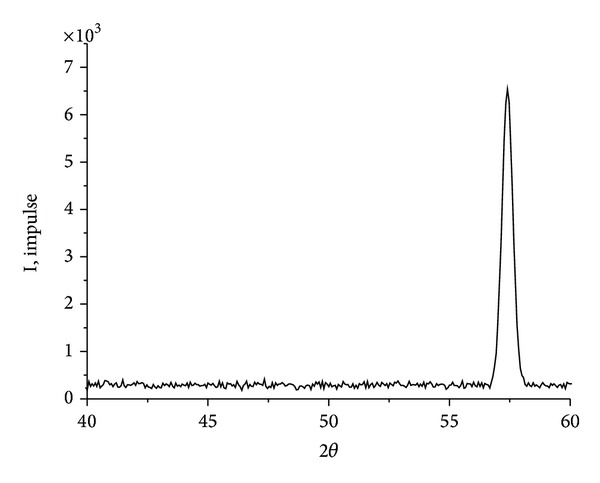
XRD spectrum of iron samples obtained by reduction of sample 2 in hydrogen flow at 400°C.

**Figure 3:**
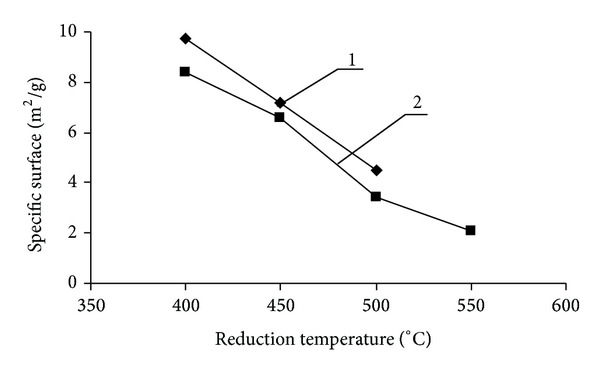
The dependence of the specific surface of iron on reduction temperature: (1) iron powder obtained from FeOOH dried at 25°C, (2) iron powder obtained from FeOOH dried at 110°C.

We presumed that for obtaining of metal particles with high dispersion need to reduce their oxides at lower temperatures [2]. This fact demands that we have to approach more carefully to the selection of the original matrix. We felt it appropriate to use the amorphous iron hydroxide (*α*-FeOOH). Iron hydroxide (*α*-FeOOH) and hydrogen with purity of at least 99.99% are used as starting substances. *α*-FeOOH was prepared by precipitation of salt FeSO_4_·7H_2_O, NaOH is used as a precipitant, and distilled water is used as solvent. The resulting precipitate of hydroxides was washed with distilled water and dried. During the heating at 200°C the pyrolysis of iron oxyhydroxide (III) (FeOOH) carried out to form Fe_2_O_3_. Specific surface (Ssp) for iron hydroxide determined by nitrogen adsorption (BET) is 198 m^2^/g. Recalculation of experimental values of the specific surface S was carried out by the formula: dav = 6/*ρ*S, (1), where *ρ* is density and S is surface area. The formula allows us to estimate the average particle size of powders dav. The calculated average size of FeOOH particles is 7 nm. According to the Mössbauer microscopy, obtained samples of iron (III) oxyhydroxide warmed at 25°C were the fine particles which follow from the spectrum form (doublet) of the investigated sample. Metal nanopowders are received by recovery of FeOOH (1 g) in a quartz tube furnace with nichrome heaters in a hydrogen atmosphere at a temperature range from 300 to 1000°C. Reduction was carried out by hydrogen with purity not less than 99.99%. The hydrogen flow rate was 2,5·10^−6^ m^3^/s. The effect of reducing conditions on the phase composition, structure and dispersion of metallic iron powder was studied. Two forms of iron hydroxide were studied: (1) hydroxide dried at a temperature of 25°C (Sample 1), (2) hydroxide dried at 110°C (sample 2) (Table 1). The identification of the powder phases was performed by X-ray powder diffraction on a diffractometer Bruker “D2 Phaser” with a cobalt anode. XRD of metal iron powder after reduction for 90 minutes at 450°C (Figure 2) and after reconstitution for 60 minutes in the temperature range 500–1000°C showed that the samples contain only *α*-Fe. Comparison of the iron nanoparticle sizes of FeOOH samples, (heat-treated at 25°C and 110°C) and reduced at different temperatures, show (Figure 3) that increasing in reduction temperature from 400 to 550°C increases the size of metallic iron particles. It should be noted that for samples FeOOH heat-treated at 25°C and reduced at the temperature range 400–550°C the obtained resulting iron nanoparticles are higher than those nanoparticles obtained by reducing of samples FeOOH heat-treated at 110°C. At reduction temperatures 450–500°C, a dramatic increase in the size of nanoparticles of metallic iron with their transition to micronscale occurs. This fact we attributed to the process of sintering of metal particles. It was revealed that for reduction in the temperature range 400–450°C of initial iron hydroxides, iron nanopowders with an average particle size of 61–81 nm can be prepared, representing the phase of *α*-Fe.

Thus, it can be assumed that the production of dispersed iron hydroxides by heat treatment of initial iron hydroxides in a reducing atmosphere is an optimal process because it allows to obtain a dispersed metal in comparison with the metal reduced from oxide.

Study of uniaxial compaction of iron powders with a particle size of 60 nm obtained by reduction of iron hydroxide in a hydrogen atmosphere was carried out in a cylindrical mold having an inner diameter 15 mm; compact height was 5–7 mm. Pressure was varied from 0.05 to 1.1 GPa. The density of compacts was determined by hydrostatic weighing with an accuracy of 2%. The most intense compaction of powders occurs at pressures of up to 0.8-0.9 GPa.

As a result, the optimal pressing conditions were determined as follows for iron nanopowders weight of powder—9 g, size of obtained sample: height (*h*)—4,96 mm, diameter—15 mm, *ρ* = 4,60 g/cm^3^, applying pressure of 200 MPa for 60 seconds, holding for 180 sec, and discharge for 30 seconds.

As a result, the regulation conditions of residual porosity of metallic sample were found in the range from 1 to 35%. Such porosity is required for the carrying out of surface chemical reactions on the iron particles surface.

Synthesis of titanium-carbon groups and titanium-carbon nanostructures on the iron surface was carried out on the hydroxylated surface of samples by ML-ALD. In this paper objects of study on which we investigated the surface chemical reactions were: (1) modeling samples—monocrystalline silicon wafer (KDB-7, 5 with orientation (100)) with deposited layer of *α*-Fe with size 1.5 × 1.5 cm and iron film thickness of ~100 nm and (2) dispersed iron particles obtained by the method described above; dispersed iron nanoparticles are characterized by the following parameters: a particle size of 40–80 nm, specific surface area of 6 m^2^/g.

Since the surface of the iron particles is always oxidized and thickness of the oxide layer is not known, then all the investigated objects before the synthesis are treated (chlorinated) by vapor CCl_4_ at 350°C to remove the oxide layer. Interaction reactants with the iron powder surface by ML-ALD were performed in a flow-type reactor under dry inert gas flow (helium) which simultaneously ensures removal of gaseous reaction products from the reactor. For the analysis of chemical reactions on the silicon plates with iron layer we used thermogravimetric setting in which analytical electronic scales BP 221S were used to register the change in sample weight. Quartz cup with the test substance (silicon plate with a layer of iron) is attached to scales with quartz fiber. In some experiments method of chemical vapor deposition (CVD) is used to obtain titanium carbide from the gas phase.

To study the mechanical strength of the samples of metal (iron-based) composite materials nanostructured titanium carbide, ultimate stress limit-ultimate resistance (*σ*_*v*_) is determined on desktop testing machine AG-50kNXD (Shimadzu) in the resource center of innovative technologies of composite materials (St. Petersburg State University).

## 3. Results and Discussion

Synthesis of titanium-carbon groups and titanium-carbide nanostructures was performed by ML-ALD on chemically prepared surface of iron sample, that is, on a surface which contains reactive functional groups (OH, OCH_3_, H, Cl, or others).

### 3.1. Experimental Substantiation of the Synthesis of Titanium-Carbon Nanogroups and TiC Nanostructures on the Iron Particles Based on the Study of Surface Reactions of Functional Groups with Low-Molecular-Weight Reagents

In the study objects, on which the surface chemical reactions were studied are

1. the model samples-plates of single-crystal silicon with deposited layer of *α*-Fe with thickness of ~100 nm;
2. dispersed iron particles obtained by the method described above; nanoscale dispersed iron particles characterized by the following characteristics: a particle size of 40–75 nm, specific surface of the particles—6 m^2^/g;
3. porous pressing from iron particles as starting intermediates for the synthesis of titanium-carbon nanolayers in the pores of the metal matrix.

#### 3.1.1. Obtaining of Nanolayer of Titanium-Carbon Groups by ML-ALD on Silicon Sample Coated with Iron Nanolayer

To clarify the mode of deposition of titanium carbide nanolayer, model samples representing monocrystal silicon wafer with a sputtered layer of pure iron were used.

When carrying out the surface chemical reactions their behaviors are dependent on temperature and holding time in the reaction zone. Note that the reaction occurs in the range 300–700°C. Our proposed method of synthesizing of titanium carbide by ML-ALD is based on reactions of chemical condensation (1)–(4) according to the following scheme:

(1) $$\begin{array}{c} \equiv \text{Si}\cdots {\left[\text{Fe}\right]}_{\text{a}}\text{H}+{\text{CCl}}_{4}⟶\;\equiv \text{Si}\cdots {\left[\text{Fe}\right]}_{\text{a}}\text{–Cl}+\text{HCl}↑ \end{array}$$

(2) $$\begin{array}{c} \equiv \text{Si}\cdots {\left[\text{Fe}\right]}_{\text{a}}\text{–Cl}+{\text{CH}}_{4}⟶\;\equiv \text{Si}\cdots {\left[\text{Fe}\right]}_{\text{a}}\text{–}{\text{CH}}_{3}+\text{HCl}↑ \end{array}$$

(3) ≡Si⋯[Fe]a–CH3+TiCl4 ⟶ ≡Si⋯[Fe]a–CH=TiCl2+HCl↑

(4) ≡Si⋯[Fe]a–CH=TiCl2+CH4 ⟶ ≡Si⋯[Fe]a–CH=Ti–(CH3)2+HCl↑

where *≡*Si*⋯*[Fe]_a_H represents surface atoms of silicon plates with a deposited film of pure iron*⋯*[Fe]_a_ with thickness of ~10 nm and containing surface hydrogen atoms (H) after treatment by hydrogen. Bond *≡*Si*⋯*[Fe]_a_H denotes an adhesive connection with a layer of silicon with iron layer. The product of 1 cycle of reactions ML-ALD—*≡*Si*⋯*[Fe]_a_–CH=Ti–(CH_3_)_2_—contains titanium-carbon groups (–CH=Ti–(CH_3_)_2_) associated covalent bond with surface methyl sites capable to reacting with TiCl_4_ (see scheme in Figure 7).

The synthesis was carried out on silicon wafers with a sputtered film of pure iron with thickness of ~10 nm. The sample was alternately treated at 500°C in a flow-type reactor by carbon tetrachloride and methane.

**Figure 4:**
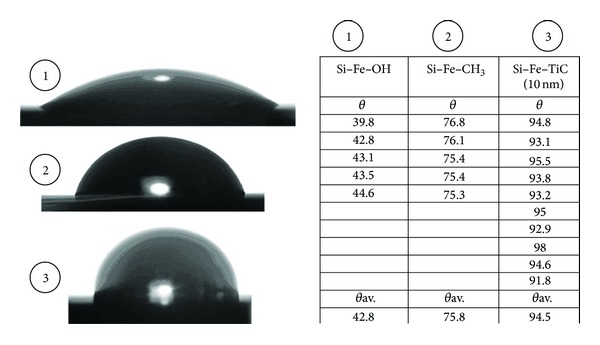
The values of contact angles by water for silicon samples with iron layer with different functional groups: (1) *≡*Si–Fe–OH, (2) *≡*Si–Fe–OCH_3_, and (3) *≡*Si–Fe–TiC.

Cycle is repeated again when thickness increasing is needed. Then the reactor with sample was cooled in the hydrogen flow. To determine the coating continuity of functional groups (–CH_3_) on the iron surface the water contact angles were studied. The study of contact angle was conducted by photomicrograph method. Based on a study of water contact angle of samples (initial silicon with hydroxyl groups, silicon with methyl groups and silicon with deposited titanium-carbon layers), regular increase of water contact angle from 43° to 76° and 94° was identified which indicates the transition to higher packing density of surface groups in the case of reactions involving methyl (–CH_3_) group (Figure 4). Study of reactions (1)–(4) was performed in the temperature range 200–600°C.

**Figure 5:**
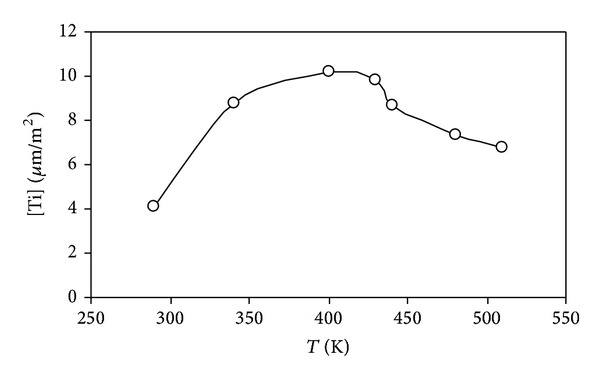
The dependence of Ti content in nanolayer from synthesis temperature.

Data on the chemical analysis of titanium have concluded (Figure 5), that at low temperature synthesis there is observed an increase of titanium and chlorine content which reach maximum at about 350–400°C, the amount of reacted titanium and chlorine in the sample remains approximately constant and the ratio of Cl/Ti is 2,6-2,7. Above 450°C, a significant slope in the titanium content and a high chlorine concentration on the surface are observed.

Decline of the titanium content at warming temperature of 450°C can be associated with the thermal decomposition of surface groups. Apparently, in these conditions the interaction TiCl_4_ with the surface leads to dissociative process: TiCl_4_ → TiCl_3_ + Cl_2_, resulting in chlorine chlorinating the surface to form complex C–Cl that blocks it from further reaction with TiCl_4_.

Macrohardness study and thin section structures of the synthesized samples (Table 2) also showed that the optimal synthesis temperature is 400°C.

The study of dependence of titanium content on silicon samples with metallic iron layer from treatment showed that the growth dependence of the titanium content is linear and after 5-6 cycles the titanium content increasing becomes constant. Note that the optimum concentration of the hydrogen significantly increases the rate of titanium carbide formation. This is probably due to the suppression of the reaction of free carbon formation.

#### 3.1.2. Production of Titanium Carbide Micron- and Nanolayer on Iron Nanoparticle Surfaces

Obtaining of titanium carbide on the surface of dispersed iron was carried out on particles with size 40–80 nm and specific surface area 6 m^2^/g. Treatment of iron powder in a quartz reactor was performed in a flow of dry argon.

To study the thickness effect of the deposited layer of titanium carbide on the mechanical properties of the samples, obtaining of ultrafine micron films of titanium carbide by chemical vapor deposition (CVD) and nanolayers of titanium carbide by atomic layer deposition (ML-ALD) was investigated. Treatment of iron powder was performed in a quartz reactor in dry argon flow.

**Figure 6:**
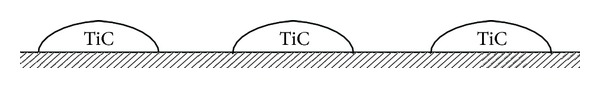
Structure of the samples after chemical deposition (CVD) of titanium carbide at sequential supplying of titanium tetrachloride and methane mixture.

In gas phase deposition of titanium carbide formation of continuous frame in the pores of metal workpiece begins with the formation of island structures on the surface. This character of the formation is associated with the feature of the reaction of chemical deposition of titanium carbide as surface is energetically heterogeneous. This leads to different rates of growth of the titanium carbide on different sites and forming island structures—three-dimensional nucleus of titanium carbide [6] (Figure 6).

Feature of nanolayers obtained by ML-ALD is the absence of phase-formation (nucleation) under maintenance of certain synthesis conditions which allows more fine-tune of the functional properties of the resulting material.

As a result of the synthesis there were synthesized examples of iron powder with different quantity of titanium carbide deposited on the surface. The content of titanium-carbon groups in the sample was determined by the titanium content. Analysis of titanium and carbon was performed by chemical method (photometrically). Data of the chemical composition of samples are given in Table 3.

Observance of conditions of ALD reactions allows obtaining monolayers with certain chemical composition; the number of performed reactions controlled the thickness of the deposited layer of substance. Features of layers produced by chemical assembly are the absence of phase formation (nucleation) under certain synthesis conditions which allows to regulate more fine the functional properties of the obtained material.

**Figure 7:**
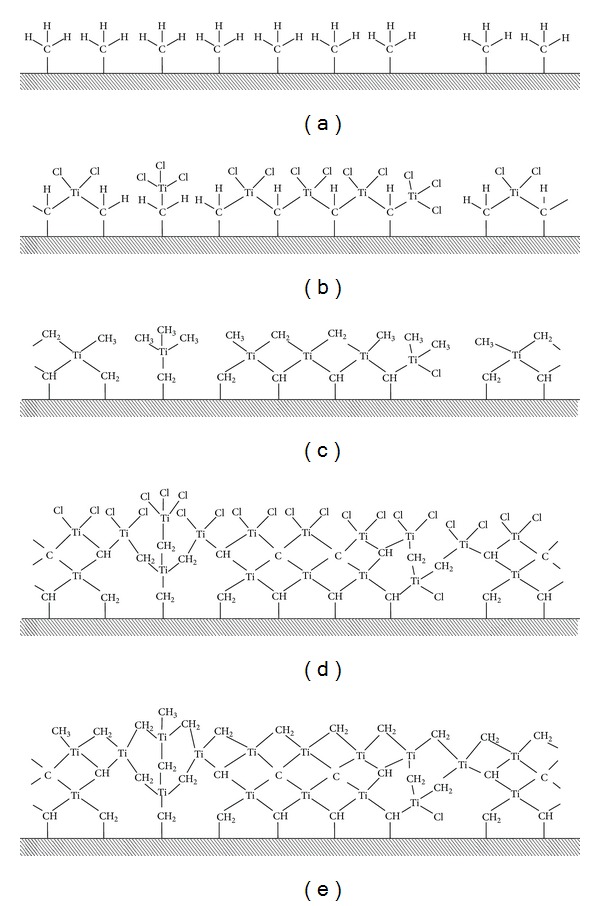
Diagram of the samples after process of atomic layer deposition with the cyclic supply of titanium tetrachloride and methane. (a) Initial surface of dispersed iron particles with methyl functional groups after exposure CH_4_; (b) exposure of titanium tetrachloride giving titanium-chloride groups; (c) exposure of methane giving titanium-carbon groups; (d) and (e) repeated sequential exposures of titanium tetrachloride and methane, respectively (reactions (1)–(4)).

Figure 7 shows a scheme of nanolayer structure of titanium-carbon groups. Features of the structure of nanocrystalline materials (grain size, a significant proportion of the interfaces and their state, porosity, and other structural defects) are defined by methods of their production and have a significant influence on their properties. With decreasing of grain size, strength increase with maintaining of ductility, effect of low temperature, and high superplasticity are manifested; a change in physical properties is observed.

As it follows from Figure 7 the repeated sequential exposures with titanium tetrachloride and methane, respectively, lead to the growth of amorphous nanolayer of titanium-carbon groups. Note that as following from the XRD data samples after synthesis at 400–500°C were amorphous.

**Figure 8:**
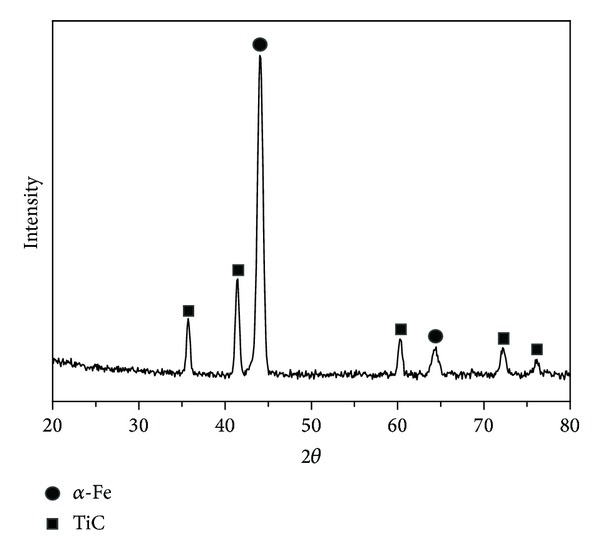
XRD spectrum of samples of dispersed iron with titanium-carbon nanostructures after treatment with 10 cycles of ALD reactions and sintered at 1100°C.

From the X-ray diffraction analysis data (Figure 8) there follows that nanostructures of TiC are formed on iron particle surface after 10 cycles of surface reactions and calcination at 1100°C for 5 hours. Considerable interest to bulk nanocrystalline materials was determined from the fact that their structural and functional properties differ significantly from the properties of coarse-grained analogues.

Analysis of the compaction and sintering of metallic iron samples nanostructured by nanowires TiC allows finding the conditions for obtaining metallic samples with low porosity. To achieve the lowest porosity (5-6%) the sintering of compacts is necessary to be carried out by heating from 500 to 800°C for 2 hours with intermediate equalizing at 800°C in hydrogen for 3 hours.

### 3.2. Study of Mechanical Properties of Nanostructured Bulk Metal (Iron-Based) Composite Material with Structural Nanoheterogeneities Based on TiC

Mechanical properties of nanomaterials essentially depend on the grain size. At large grain sizes a strength and hardness growth with decreasing grain size is determined by introduction of additional grain boundaries that are obstacle to dislocation motion and at small nanoscale grains a strength growth is determined by low density of existing dislocations and the difficulty of formation of new dislocations [2].

To study the mechanical strength of the samples of metal (iron-based) composite materials nanostructured titanium carbide ultimate stress limit-ultimate resistance (*σ*_*v*_) is determined. These studies are presented in Table 4. For comparison the table shows the best mechanical properties of the steels according to [37]. Table 4 shows that the mechanical properties of the iron-based composite materials with TiC dispersed phase, despite the presence of residual porosity, are comparable with the best properties of steel grades which contain many dopants.

It should be borne in mind that the samples have porosity, that allows to improve the mechanical properties of specimens.

Thus we can assume that the creation of metallic (iron-based) composite materials nanostructured titanium carbide using surface structuring process is promising for producing of new generation composite materials.

## 4. Conclusions

Through a nanostructuring process of metal (iron) matrix developed by authors the synthesis of samples of nanostructured metal matrix composite with nanostructural (1–50 nm) inhomogeneities on the basis of TiC with the crystal structure in the volume of the iron matrix was reformed.

The synthesis, pressing, and sintering conditions of dispersed iron nanoparticles (40–75 nm) with a surface area of 6 m^2^/g were investigated.

The features of the behaviour of surface chemical reactions of functional groups on the metallic iron surface with low molecular substances (TiCl_4_, CH_4_) were studied and it was found that methyl (CH_3_) groups are most suitable for the synthesis of carbide nanostructures in inert environment compared with hydroxyl (OH) or methoxy (OCH_3_) groups.

A method of synthesis of titanium carbide nanostructures on the surface of dispersed iron particles using methyl (CH_3_) functional groups, TiCl_4_, and CH_4_ is developed.

The sequence of synthesis steps of titanium-carbon nanostructures on the surface of the dispersed iron particles, concentration and temperature ranges at which the synthesis is carried out, and the temperature intervals of sample heating were established. Based on X-ray analysis it is shown that in a cyclic process of exposure of TiCl_4_ vapor and CH_4_ on dispersed iron titanium-carbon nanolayer with amorphous structure is formed at the surface. Crystallization and the formation of titanium carbide are carried out after calcinations at 1100°C.

Investigation of sintering and pressing conditions of dispersed iron particles with deposited titanium carbide nanostructures allows to determine the conditions for obtaining a metal (iron-based) composite material with low residual porosity with a dispersed phase based on titanium carbide.

Mechanical properties of the metal (iron-based) composite material with dispersed phase based on titanium carbide are studied and it is found that it is comparable with the best grades of steel obtained by moulding.

The results suggest that the creation of metallic (iron-based) composite materials structured by titanium carbide nanostructures using the process of surface structuring is promising for the preparation of a new generation of composite materials.

## Figures and Tables

**Table 1:** Specific surface and average size of aggregates of fine dispersed iron reduced at different temperatures.

Initial oxide	Reducing temperature, °C	*S*_sp_, m^2^/g	*D*_av_, 10^−9^ m
FeOOH dried at 25°C	400	9,7	82
	450	7,2	109
	500	4,5	173

FeOOH dried at 110°C	400	8,4	91
	450	6,6	116
	500	3,7	206
	550	2,1	381

**Table 2:** Macrohardness and characterization of the microstructure of samples obtained by alternating exposures of titanium tetrachloride-methane.

*T*, °C	Number of cycles of ALD reactions	HV, kg/mm	Description of microstructures
200	1	84	Absence of reaction by XRD, nonuniform film
	2	82	
	4	150	
	5	144	
	6	210	

300	1	80	Absence of reaction by XRD, nonuniform film
	2	63	
	4	120	
	5	380	
	6	465	

400	1	340	Uniform fine-grained structure
	2	303	
	4	690	
	5	1302	
	6	1280	

450	1	684	Uniform fine-grained structure
	2	905	
	4	1010	
	5	1260	
	6	1200	

500	1	204	The appearance of chlorine excess which is manifested in the nonuniform structure
	2	660	
	4	785	
	5	550	
	6	930	

550	1	340	Increasing heterogeneity of structure centers
	2	280	
	4	833	
	5	535	
	6	650	

**Table 3:** Chemical composition of samples based on the iron particles with titanium-carbon groups.

Sample	Number of cycles processing	Composition of the sample	Estimated thickness (nm)
1	—	100% Fe	—
2	1	95,5% Fe + 1,5% TiC	0,5
3	2	97% Fe + 3% TiC	1
4	10	90,5% Fe + 12,5% TiC	5
5	CVD	84,5% Fe+ 19,5% TiC	15
6	CVD	74,5% Fe + 35,5% TiC	40

**Table 4:** The study of mechanical properties of the synthesized composite materials based on iron with dispersed phase of TiC.

Sample	Composition	Tensile strength, *σ*_*B*_, MPa
Obtained samples		

MN-ALD method		
1	100% Fe	183
2	95,5% Fe + 1,5% TiC	800
3	97% Fe + 3% TiC	1130
4	90,5% Fe + 9,5% TiC	1370
5	84,5% Fe + 15,5% TiC	1460

CVD method		
6	69,5% Fe + 30,5% TiC	890
7	61,5% Fe + 38,5% TiC	900

Literature data [37]		
7	Steel AS 14CGN	1120
8	Steel 50CG	1300
9	Steel 12C2N4A	1130
10	High-strength steel	1500

*The residual porosity of the synthesized samples was 5–6 %.
